# Supplementary material for: Polar coupling enabled nonlinear optical filtering at MoS2/ferroelectric heterointerfaces
Source: Nat Commun. 2020 Mar 17;11:1422. doi: 10.1038/s41467-020-15191-2 (PMC7078226; doi:10.1038/s41467-020-15191-2)
Supplement: Supplementary file 1 — Supplementary Information [file 41467_2020_15191_MOESM1_ESM.pdf]

SUPPLEMENTARY INFORMATION

Polar Coupling Enabled Nonlinear Optical Filtering at MoS_2/Ferroelectric Heterointerfaces

Li et al.

Supplementary Note 1: Characterization of $PbZr_{0.2}Ti_{0.8}O_3$ Thin Films

We deposited epitaxial $PbZr_{0.2}Ti_{0.8}O_3$ (PZT) thin films on $La_{0.67}Sr_{0.33}MnO_3$ (LSMO) buffered (001) $SrTiO_3$ (STO) substrates using off-axis radio frequency magnetron sputtering, and characterized their structural properties using x-ray diffraction (XRD). Supplementary Figure 1a shows the XRD θ-2θ scan taken on a PZT/LSMO heterostructure, which reveals (001) growth for PZT with the *c*-axis lattice constant of ~4.17 Å and no appreciable impurity phases.

We examined the polarization switching characteristics of the PZT films via piezoresponse force microscopy (PFM). Supplementary Figure 1b shows the PFM switching hystereses taken on a 50 nm PZT/10 nm LSMO sample, which reveal coercive voltages of about +2 V (-3 V) for the P_{up} (P_{down}) state. We used ±7 V DC bias voltage for domain writing and 0.5 V AC voltage for domain imaging (Supplementary Figure 1c-d). The as-grown PZT film is uniformly polarized in the P_{up} state.

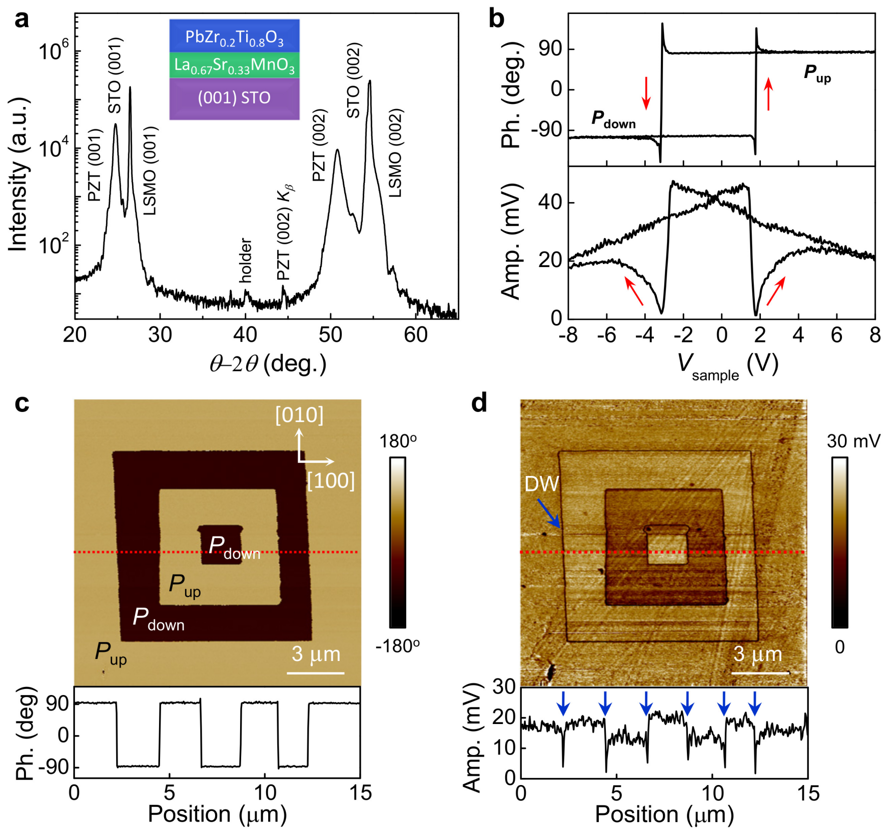

Supplementary Figure 1 | Characterization of PZT thin films. a, XRD θ-2θ scan of a PZT/LSMO heterostructure grown on (001) STO. The Laue finite size fringes are clearly visible around the Bragg peaks of LSMO. **b,** Switching hystereses of PFM phase (top) and amplitude (bottom) responses taken

on a PZT/LSMO sample with sample bias and the AFM tip grounded. **c**, PFM phase and **d**, amplitude images of the domain structure on PZT shown in Fig. 1c in the main text. The lower panels show the signal profiles along the dotted lines. The blue arrows point to the DW positions.

Supplementary Figure 2 shows the intensity of the second harmonic generation (SHG) signal taken on a PZT domain wall (DW) as a function of the excitation laser power, which exhibits a quadratic power-dependence, consistent with the second-harmonic emission. As shown in Supplementary Figure 3, the SHG response can only be detected when the analyzer orientation can be projected along the direction perpendicular to the DWs. The SHG signals detected in the reflection (Supplementary Figure 3a-d) and transmission (Supplementary Figure 3e-h) modes exhibit qualitatively similar behaviors.

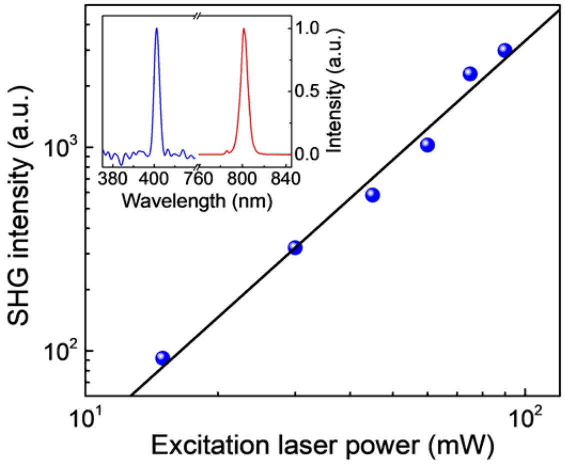

Supplementary Figure 2 | Power-dependence of the SHG response of DW in PZT. SHG intensity taken on a DW as a function of the excitation laser power (blue dots). The solid line shows the quadratic dependence. **Inset**: Normalized spectra of the SHG from the DW (blue) and the excitation laser beam (red).

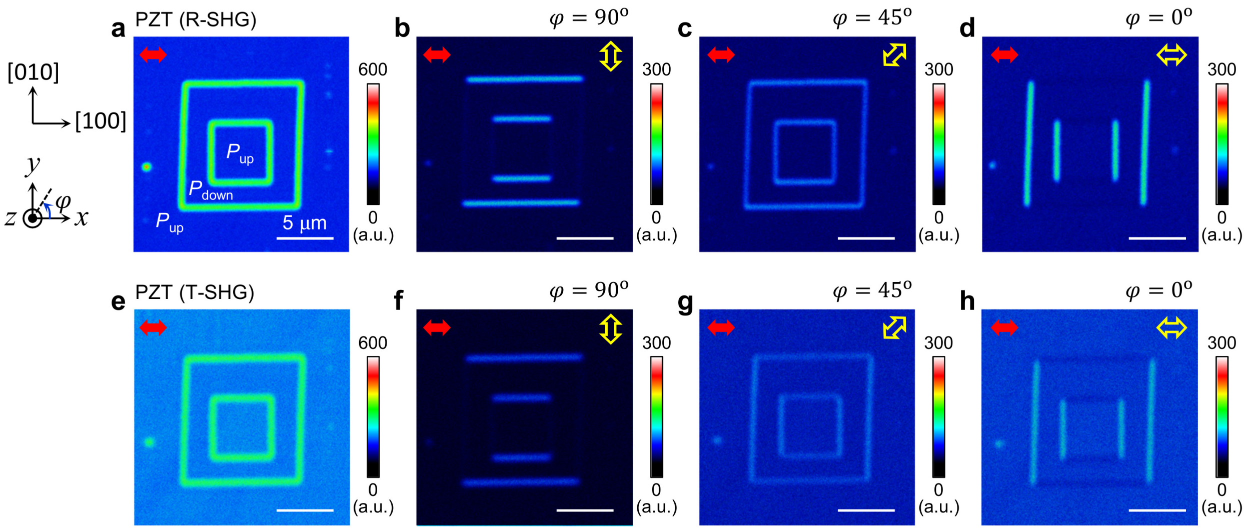

Supplementary Figure 3 | SHG mapping of square domains in PZT. a-d, SHG mapping in reflection mode (R-SHG) of square domains written on a PZT thin film taken **a,** with no analyzer applied, and **b-d,** with the analyzer applied at different angle φ (yellow open arrows) with respect to the polarizer (red solid arrows), which is along *x*-axis. **e-h,** SHG mapping in transmission mode (T-SHG) of the same domain structure taken with the same polarizer and analyzer settings as in **a-d**, respectively. The excitation laser power is 30 mW. The scale bars are 5 μm. The crystalline orientation of PZT and the laboratory coordinate system are shown as insets.

Supplementary Note 2: Preparation and Characterization of Monolayer MoS_2 on $PbZr_{0.2}Ti_{0.8}O_3$

To prepare the MoS_2/PZT heterostructure, we first mechanically exfoliated MoS_2 flakes from a bulk single crystal on a Gel-Film (Supplementary Figure 4a), and identified the monolayer (1L) flakes via Raman spectroscopy studies. The crystalline orientation of the selected flake was determined using polarized SHG measurements. As shown in Supplementary Figure 4b, we aligned the polarizer and analyzer with the top edge of 1L MoS_2 (*x*-axis), and then collected the SHG signal with the sample rotating within the *x*-*y* plane from angle $\phi = 0°$ to $360°$ in $10°$ steps. The angular-dependence of the SHG signal exhibits a characteristic six-fold rotational symmetry that can be well fitted by $I_{\text{SHG}} = I_{\max} sin^2(3\phi)$,[1,2] where $I_{\max}$ is the maximum SHG intensity, indicating that the top edge of the 1L flake is along the a-axis of MoS_2 (Supplementary Figure 4a). SHG imaging shows that the sample exhibits similar SHG response in the reflection (Supplementary Figure 4c) and transmission (Supplementary Figure 4d) modes.

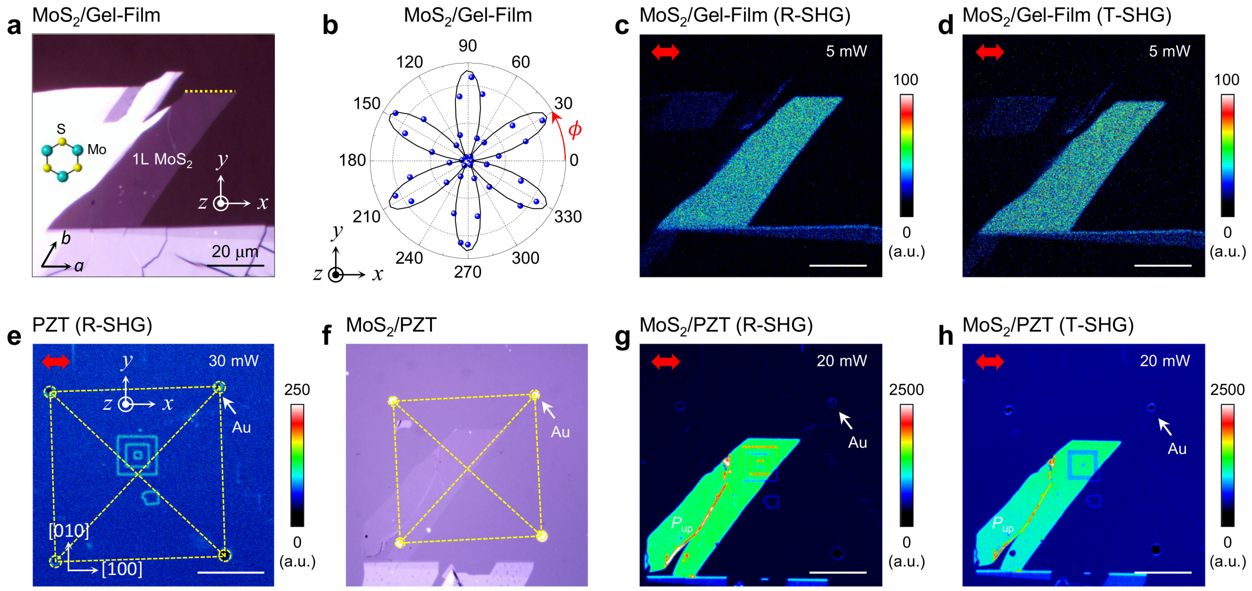

Supplementary Figure 4 | Fabrication of 1L MoS_2/PZT heterostructure. a, Optical image of a MoS_2 flake exfoliated on a Gel-Film. The dotted line marks the top edge of the 1L region. **b,** Polar plot of the parallel SHG intensity taken on the 1L region (blue dots) as a function of the sample rotation angle ϕ with a fitted angular dependence (black solid line). **c**-**d**, SHG mapping of the MoS_2 sample on Gel-Film in **c**, the reflection and **d**, transmission modes. **e**, SHG mapping of a PZT film patterned with square domains. Also visible in the image are small Au marks (50 μm separation) deposited on PZT for locating the domain position. **f**, Optical image of the PZT sample after the 1L MoS_2 flake shown in **a-d** was transferred on top of the domain structure. There is a crack forming during transfer. The dashed lines in **e-f** serve as the guide to the eye. **g-h**, SHG mapping of the same region taken in **g**, the reflection and **h**, transmission modes. All SHG mappings were taken with the incident light polarization aligned with *x*-axis (red solid arrows) and no analyzer applied. The excitation laser powers are labelled on the images. The crystalline orientations of MoS_2 and PZT and the laboratory coordinate system are shown as insets.

We then transferred the 1L MoS_2 flake on a PZT film patterned with P_{up} and P_{down} square domains using the dry-transfer approach outlined in Ref. [3]. As shown in Supplementary Figure 4e, the PZT films have pre-deposited Au marks (50 μm apart), which can be used to locate the domain position and facilitate the alignment of the MoS_2 crystalline axis with the PZT DWs. During transfer, we aligned the *a*-axis (zigzag orientation) of MoS_2 with the horizontal DWs ([100]) of PZT (Supplementary Figure 4f). After transfer, we used SHG mapping to check if we have achieved the desired alignment (Supplementary Figure 4g-h).

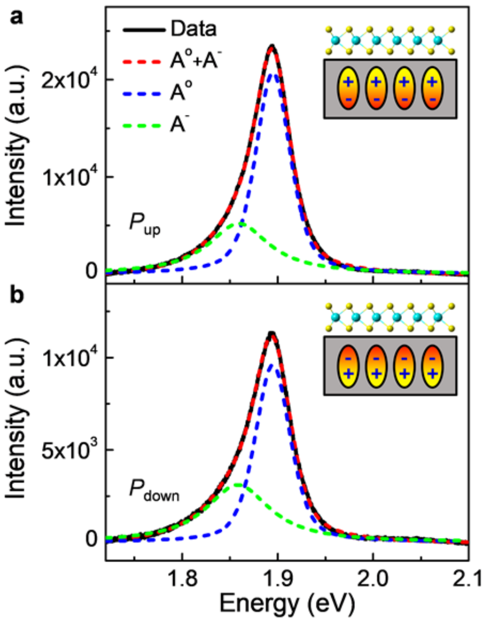

Supplementary Figure 5 | Effect of ferroelectric polarization on PL in 1L MoS_2. a-b, PL spectra of the 1L MoS_2 on the **a,** P_{up}, and **b,** P_{down} domains. The black solid lines are experimental data. The dashed lines are fits (red) by summing the A° (blue) and A^- (green) exciton contributions. **Insets:** Sample schematics.

Supplementary Figure 5 analyzes the room temperature photoluminescence (PL) spectra of the 1L MoS_2 shown in Fig. 1e. We quantitatively fitted the PL spectra using Lorentzian model with two peaks associated with the neutral exciton (A°) (blue) and negative trion (A^-) (green). The intensity areal ratios between the A^- and A° peaks are 0.54 and 0.71 for the regions on the P_{up} and P_{down} domains, respectively. Similar modulation of the PL response by the polarization of a neighboring ferroelectric layer has been observed in various transition metal dichalcogenides (TMDCs), including 1L WS_2,[4] $MoSe_2$ and WSe_2,[5] which has been attributed to ferroelectric polarization induced doping effect. Our observation, however, suggests a lower electron level in MoS_2 on the P_{up} domain, which is opposite to that expected from direct doping of the polarization field and those observed in Refs. [4,5]. This discrepancy can be understood by taking into account the presence of a surface screening layer on PZT. It is known that the surface potential of freshly polarized ferroelectric surface, when exposed to the ambient, diminishes with time[6,7] and can even reverse sign[7,8] upon temperature variation. This effect originates from the formation of a screening layer on PZT surface, *e.g.*, from charged adsorbates such as dissociated

water molecules.[9] Its slow dynamics competes with the pyroelectric effect induced polarization change, which can lead to a reversal of ferroelectric surface potential. As a result, the doping change in the TMDC layer only corresponds to the residue polarization field, *i.e.*, the under-screened or over-screened fraction, and the relative doping level depends on the preparation details of the TMDC/ferroelectric heterostructures, such as the time interval between domain patterning and TMDC transfer, the transfer temperature, speed, and environment (dry vs. wet), *etc*.

Supplementary Note 3: Second Harmonic Generation Responses of $MoS_2/PbZr_{0.2}Ti_{0.8}O_3$ Heterostructures

To clarify the observed tailoring effect of the SHG signal has an interfacial or bulk origin, we investigated how the SHG response varies with the layer thickness of MoS_2 and PZT. First, we mechanically exfoliated a MoS_2 flake with 1L, 2L, 3L, 4L, 5L, and thick multi-layer (ML) regions (Supplementary Figure 6a), as identified by the Raman spectra (Supplementary Figure 6b). We then transferred this flake on a PZT film patterned with a series of rectangular domains (Supplementary Figure 6c). Upon transfer, the *a*-axis of MoS_2 (zigzag orientation) is aligned with the long side of the domains ([100] in PZT). Supplementary Figure 6d shows the reflected SHG mapping of the MoS_2/PZT heterostructure with no analyzer applied. We observed the following features in this data:

1. The 1L region (right part of the flake) exhibits alternately enhanced or suppressed SHG signal along the horizontal ([100]) DWs, consistent with that in Figs. 2-3 in the main text.
2. The 3L (upper-right) and 5L (center) regions of the flake exhibit similar alternating enhancement and suppression of the SHG signal along the horizontal DWs, consistent with the pattern observed on the 1L flake. The overall signal strength is lower in thicker flakes.
3. For the 2L (upper-left) and 4L (top) regions of the flake, there is no SHG signal emerging from MoS_2. Weak SHG signals were observed at the PZT DWs, which is consistent with that of bare PZT.
4. In the ML region (lower part), the SHG signal from PZT is attenuated with MoS_2 layer thickness, and eventually diminishes.

The fact that the DW tailoring effect is only observed in odd-layer thin MoS_2 flakes but absent in the even-layer MoS_2 demonstrates that it indeed originates from the noncentrosymmetric symmetry of MoS_2. The gradually diminishing tunning contrast in thicker MoS_2 agrees with fact that the SHG signal of MoS_2 attenuates rapidly with increasing layer numbers, confirming that it is an interfacial rather than bulk phenomenon.[2] The tailoring effect is also consistent with the coherent interference between the SH fields of the two constituent layers.

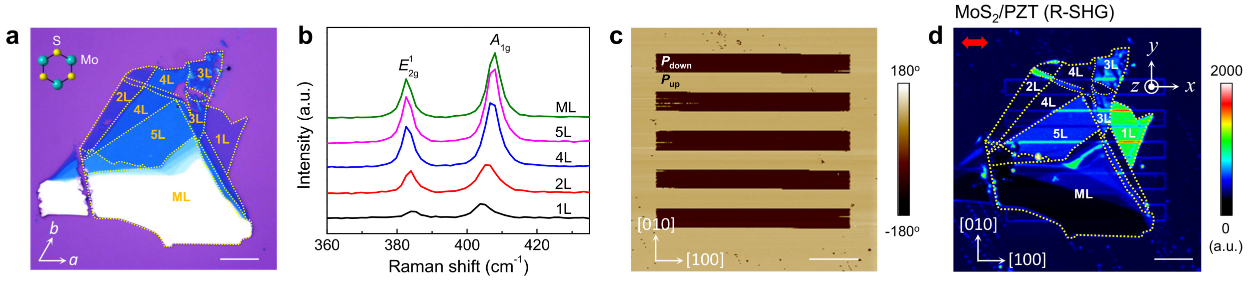

Supplementary Figure 6 | Effect of layer thickness of MoS_2. a, Optical image of an exfoliated MoS_2 flake with different layer numbers on SiO_2/Si substrate, with **b**, the corresponding Raman spectra taken on different regions. **c**, PFM phase image of a series of rectangular domains written on a 50 nm PZT thin film. **d**, Reflected SHG mapping of the MoS_2 sample in **a** transferred on top of the PZT domain structure shown in **c**. The red arrow marks the incident light polarization (along *x*-axis). The excitation laser power is 20 mW. The scale bars are 10 μm. The dotted lines serve as the guide to the eye. The crystalline orientations of MoS_2 and PZT are shown as insets.

To further investigate the role of the bulk state of PZT, we carried out the SHG measurements on MoS_2/PZT heterostructures with different PZT layer thickness. Supplementary Figure 7 compares the SHG results obtained on the heterostructures composed of 20 nm, 30 nm, and 50 nm thick PZT films. Despite the different PZT thicknesses, all heterostructures exhibit qualitatively similar SHG responses in the reflection mode, with alternating enhancement and suppression of the SHG signal at the horizontal DWs ([100]) and no SHG contrast at the vertical DWs ([010]). This result confirms that the DW tuning of the SHG signal is independent of PZT's thickness, yielding additional support to its interfacial nature.

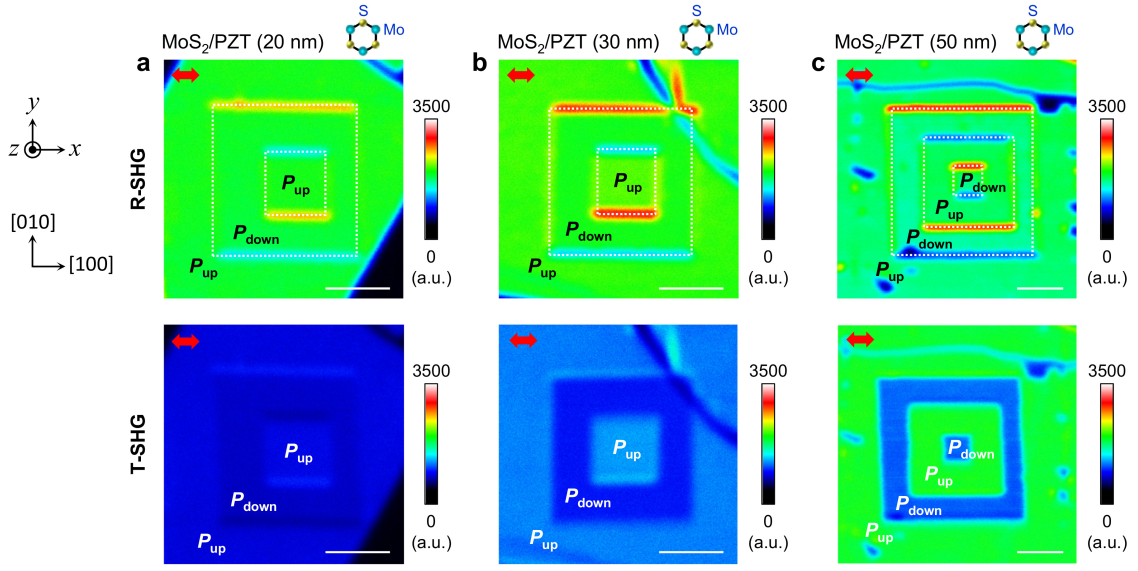

Supplementary Figure 7 | Effect of film thickness of PZT. a-c, Reflected (upper) and transmitted (lower) SHG images of 1L MoS_2 on **a**, 20 nm, **b**, 30 nm, and **c**, 50 nm PZT films patterned with square P_{up} and P_{down} domains. The crystalline orientations of MoS_2 and PZT are shown as insets. The scale bars are 3 μm. The dashed lines indicate the DW positions. The red arrows mark the incident light polarization (along x-axis). All images were taken at the excitation laser power of 20 mW.

For the transmitted SHG, we observed qualitatively similar contrast between the P_{up} and P_{down} domains on all three samples. The signal intensity, however, is significantly enhanced in thicker films (Fig. 4f in the main text), suggesting that the tailoring effect has a bulk origin. To identify the relevant dielectric layer that contributes to the tuning of the SHG signal, we investigated how the transmitted SHG signal propagates along the -z-direction. In this experiment, we first have the objectives focused at the MoS_2/PZT interface (z = 0, Supplementary Figure 8a), and then captured a series of SHG images in the de-focused condition by gradually increasing the sample-detector distance (lowering the objective position for the transmission mode detection). As shown in Supplementary Figure 8b-h, the SHG signal gradually diminishes during de-focusing. The contrast between the P_{up} and P_{down} domains is still very sharp at z = -2 μm, and becomes barely recognizable at z = -14 μm. Considering the thicknesses of PZT (50 nm) and the LSMO (10 nm) buffered STO substrate (0.5 mm), the relevant intensity of the collected transmission signal is fully attenuated in STO before reaching

air, suggesting that the tailoring effect of the transmitted SHG signal occurs within the PZT (LSMO) layer.

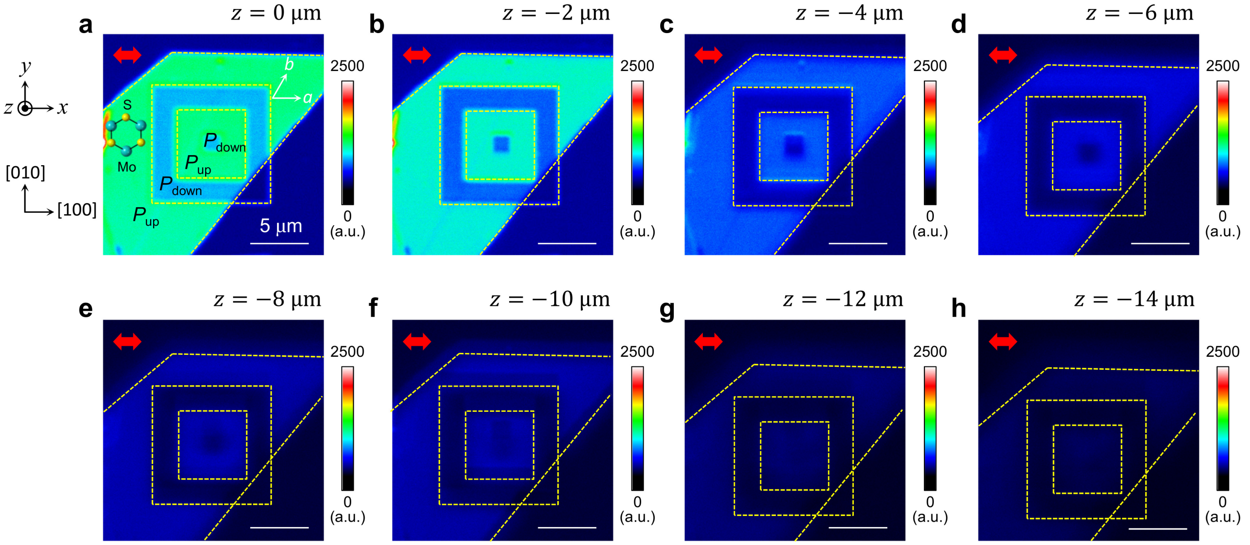

Supplementary Figure 8 | Transmitted SHG images taken on a MoS_2/50 nm PZT heterostructure at different sample-detector distance. The objective was first focused at the MoS_2/PZT interface with **a**, $z = 0$, and then **b-h**, gradually moved away from the sample by lowering the objective position (decreasing z). The dashed lines serve as the guide to the eye. The red arrow marks the incident light polarization. All images were taken at an incident laser power of 20 mW.

Supplementary Note 4: Modeling of the Polar States of Monolayer MoS_2 and Ferroelectric Domain Wall

The polar states of 1L MoS_2 and PZT DW were modeled via first-principles density functional theory (DFT) calculations, which have been performed using Quantum ESPRESSO[10] with ultrasoft pseudopotentials.[11] The exchange and correlation effects were treated within the local density approximation (LDA).[12] The plane-wave cut-off energy of 60 Ry was used in the calculations. The lattice constant and atomic coordinate were relaxed until the force on each atom was less than 0.001 eV/Å. For simplicity, the atomic structure of $PbTiO_3$ was used (a = 3.86 Å and c = 4.03 Å), whose theoretical polarization is comparable to the experimental values for $PbZr_{0.2}Ti_{0.8}O_3$.[13,14] To calculate the in-plane polarization of $PbTiO_3$, we used the theoretical bulk lattice constants of $PbTiO_3$ with polarization along the z-direction by relaxing the atomic positions, with a small initial lateral displacement of Ti along the x-axis (Supplementary Figure

9a). This approach is consistent with previous transmission electron microscopy result, indicating that the lattice constant is not changed in the flux-closure area of PZT.[15] For 1L MoS_2, periodic boundary conditions with a 20 Å cell size in the *z*-direction were used to simulate the polarization along one of the polar axes along the armchair direction (Supplementary Figure 9b). To calculate the electronic structure of $PbTiO_3$ and MoS_2, 16 × 16 × 16 and 16 × 16 × 1 *k*-point meshes in the irreducible Brillouin zones were used, respectively. Polarizations of $PbTiO_3$ and MoS_2 were obtained using the Berry phase method,[16] and the results are listed in Supplementary Table 1.

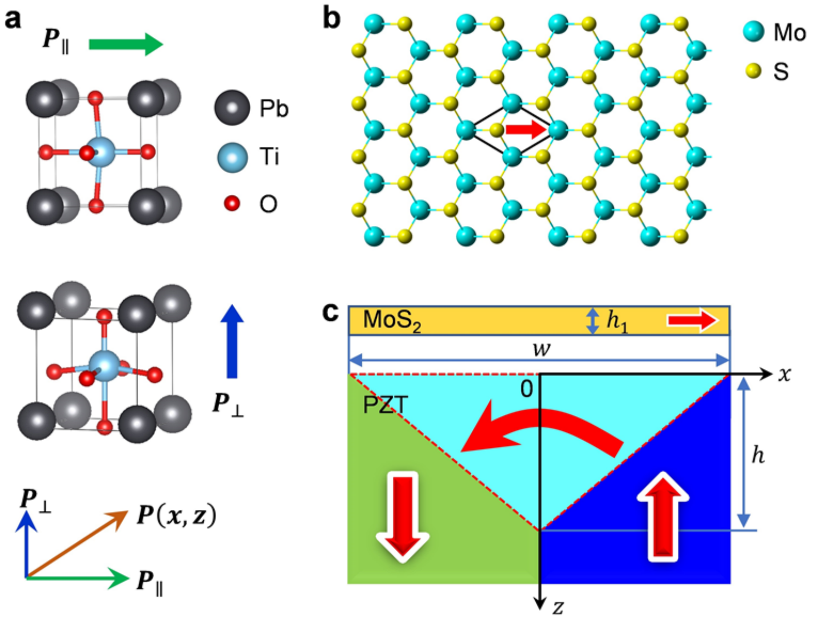

Supplementary Figure 9 | Modeling of 1L MoS_2/PZT heterostructure. a, Schematic unit cell of $PbTiO_3$ in the in-plane (top) and out-of-plane (bottom) polarization states. **b**, Schematic top-view of 1L MoS_2 lattice with the unit cell outlined. **c,** Schematic view of the polar alignment between 1L MoS_2 and a flux-closure domain (outlined by red dashed lines) in PZT. The red arrows mark the polarization orientation.

	a (Å)	*c* (Å)	*P* (μC cm^{-2})
$PbTiO_3$ (out-of-plane)	3.86	4.03	78.1
$PbTiO_3$ (in-plane)	—	—	59.1
MoS_2	3.13	—	85.5

Supplementary Table 1 | Calculated lattice parameters and polarization values for bulk $PbTiO_3$ and 1L MoS_2.

To estimate the in-plane polarization at the DW, we considered a flux-closure domain of triangular shape forming above a 180° DW near the surface of PZT (Supplementary Figure 9c), as revealed in previous transmission electron microscopy studies.[15] Within this area, there is a continuous rotation of the dipole direction from downward in the P_{down} domain to upward in the P_{up} domain. We then modeled the polarization rotation in a triangular flux-closure domain area with maximum width w along x-direction and depth h along z-direction (Supplementary Figure 9c). The polarization at point (x, z) inside this triangular area is $P(x,z) = (P_{\parallel}(x,z), P_{\perp}(x,z))$, where $P_{\parallel}(x,z)$ and $P_{\perp}(x,z)$ are the x- and z-components of polarization, respectively. At the boundary of the triangular area, corresponding to $x = \pm w(z)/2$ with $w(z) = \frac{h-z}{h} w$, the polarization is perpendicular to the MoS_2/PZT interface (*i.e.* $P_{\parallel} = 0$). In the middle of the area at $x = 0$, the polarization is in-plane (*i.e.* $P_{\perp} = 0$). Between $x = 0$ and $x = \pm w(z)/2$, we assumed that the magnitude and direction of the local polarization change linearly as a function of coordinate (x, z). Therefore, we can define $P_{\parallel}(x,z)$ and $P_{\perp}(x,z)$ as follows:

$$P_{\parallel}(x,z) = P_{\text{in}}\left(1 - \left|\frac{x}{w(z)/2}\right|\right) = \left(1 - \left|\frac{2xh}{(h-z)w}\right|\right) \cdot P_{\text{in}},$$

$$P_{\perp}(x,z) = \frac{x}{w(z)/2} \cdot P_{\text{out}} = \frac{2xh}{(h-z)w} \cdot P_{\text{out}}, \tag{S1}$$

where P_{in} and P_{out} are the in-plane and out-of-plane polarization of bulk PZT (Supplementary Table 1), respectively. The total in-plane dipole moment per unit length in the flux-closure area was calculated by integrating $P_{\parallel}(x,z)$ (Eq. 1 in the main text).

Another possible scenario for the lateral polarization $P_{\parallel}$ at the DW considers the electric field distribution of the biased AFM tip during domain writing. Supplementary Figure 10a-b show the simulated electrical potential and field distribution in PZT upon applying a bias voltage (V_{bias}) to a point contact (conductive AFM tip) using finite element analysis (Ansoft Maxwell 3D V.14). At sufficiently high electric field, it is expected that the local dipole orientation follows the electric field line direction while V_{bias} is applied. If the dipole is frozen to the same orientation after V_{bias} is removed, *i.e.*, the AFM probe is scanned away from the location, a lateral polarization can emerge at the DW. Supplementary Figure 10c shows a schematic view of a positively biased conductive AFM probe scanning from 0 to $+x$ direction, switching a P_{up} domain into P_{down} domain, resulting in a 180° DW at x = 0. It depicts such type of polar re-

orientation within the DW at $x < 0$. Note that this condition does not apply to the $x > 0$ region, which is fully polarized into the P_{down} domain by the sequential scans.

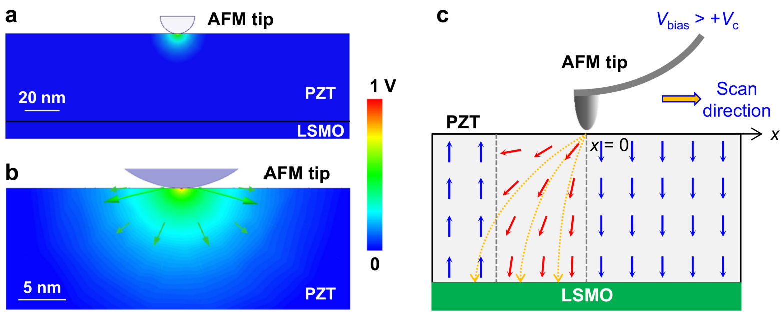

Supplementary Figure 10 | Effect of biased AFM tip on the local dipole orientation within the DW. a, Finite element analysis of the electrical potential distribution in a 50 nm PZT film with global 10 nm LSMO bottom electrode at the bias voltage of V_{bias} = 1 V applied to a point contact (AFM tip), with **b**, the expanded view close to the top surface of PZT. The arrows illustrate the simulated local electric field. **c**, Schematic view of a positively biased scanning AFM tip switching a P_{up} domain to P_{down} domain, assuming the local dipole follows the electric field line direction. Here $V_{bias} > V_c$, where V_c is the coercive voltage. The blue (red) arrows mark the dipole direction within the domain (DW). The dotted lines indicate the electric field lines when the biased AFM tip locates at $x = 0$. The dashed lines mark the boundaries of the DW.

In this scenario, the lateral polarization $P_{||}$ can persist to the bulk region of the film well exceeding 5 nm (Supplementary Figure 10b), and it points from the P_{down} domain to the P_{up} domain. This is in sharp contrast to the flux-closure model (Supplementary Figure 9c), where the chiral dipole rotation is confined within the surface 2-3 nm, and the corresponding $P_{||}$ points from the P_{up} domain to the P_{down} domain. We believe the flux-closure type configuration is more energetically favorable for two reasons:

1. In the bulk region of PZT film, the lateral dipole orientation cannot be sustained at the DW after V_{bias} is removed, as it significantly increases both the electrostatic energy (leading to charged DW) and elastic energy. This energy anisotropy between different crystalline axes is especially strong in PZT films on STO due to the large compressive (3.6%) strain.
2. At PZT surface, the second model depicts an abrupt change of polar angle, *i.e.*, close to 90° polar angle change, at the interface with the P_{up} domain (Supplementary Figure 10c), which closely resembles the emergence of *a*-domain in a *c*-axis oriented film. The 90° DW is

charged and naturally wider than the 180° DW. It has been theoretically predicted that the widths for the 90° and 180° DW are 3.6 nm and 0.6 nm, respectively.[17] This means the spatial extension of this kind of DW configuration is comparable with that of the flux-closure type DW (2-3 nm),[15] while the 90° polarization change corresponds to a much higher electrostatic energy than the gradual polar rotation.

Supplementary Note 5: Modeling of the Second Harmonic Generation Response

The SHG results in Figs. 2-3 can be well interpreted via the nonlinear electromagnetic theory. As the thickness of MoS_2 and extension of the flux-closure DW in PZT are well below the optical wavelength, the fundamental electric field is essentially the same for both layers. The SHG response of the heterointerface can be obtained by summing up the second harmonic polarization vectors from each constituent layer, so the second-order optical susceptibility tensor of the composite system ($\chi^{(2)}_{\text{interface}}$) can be directly expressed as the superposition of the terms for 1L MoS_2 ($\chi^{(2)}_{\text{MoS}_2}$) and PZT DW ($\chi^{(2)}_{\text{DW}}$): $\chi^{(2)}_{\text{interface}} = \chi^{(2)}_{\text{MoS}_2} + \chi^{(2)}_{\text{DW}}$.

The susceptibility tensor is usually replaced by a contracted *d*-tensor, which is defined as $d = \frac{1}{2}\chi^{(2)}$. The *d*-tensors for 1L MoS_2[2] and tetragonal PZT[18,19] are given in Eqs. 3-4 in the main text, respectively. For ferroelectric domains, we obtained the polarization-dependent *d*-tensors via a rotation matrix transformation:[18]

$$d_{ij}^{\text{New}} = \beta_{ij} d_{kl}^{0} \alpha_{lj}{}^{-1}, \tag{S2}$$

where d^{New} is the new *d*-tensor defined in an arbitrary coordinate system (*X', Y', Z'*), d^0 is the reference *d*-tensor defined in the crystallographic frame (*X, Y, Z*), β is the rotation matrix, and α^{-1} is the transformation matrix. In our experiment, the crystallographic axes (*X, Y, Z*) coincide with the laboratory coordinate system (*x, y, z*) and the *c*-axis of PZT ([001]) is along *z*-axis. To model the SHG data in Fig. 2, we considered a square P_{down} domain embed in a P_{up} region in PZT, with the horizontal DW ([100], ∥ *x*-axis) aligned with the *a*-axis (zigzag orientation) of MoS_2 (stacking angle $\theta = 0^\circ$). The *d*-tensors for the four DWs (Top-DW, Bottom-DW, Left-DW, and Right-DW) in PZT are given in Eq. 6 in the main text. The interfacial composite tensors at the polar domains and four DWs are given in Eqs. 5 and 7 in the main text, respectively.

To simplify the calculation, we assumed that the SHG contributions from MoS_2 and PZT DW have the same maximum intensity ($d_{MoS_2} = d_{33} \approx 0.9 d_{15} = 1$), which is reasonable given their comparable dipole moments. The interfacial composite tensors can thus be reduced to:

$$d_{\mathrm{Top-DW}}^{\mathrm{interface}} = \begin{pmatrix} 0 & 0 & 0 & 0 & 0 & 2.1 \\ 2.1 & 0 & 1.1 & 0 & 0 & 0 \\ 0 & 0 & 0 & 1.1 & 0 & 0 \end{pmatrix},$$

$$d_{\mathrm{Bottom-DW}}^{\mathrm{interface}} = \begin{pmatrix} 0 & 0 & 0 & 0 & 0 & -0.1 \\ -0.1 & -2 & -1.1 & 0 & 0 & 0 \\ 0 & 0 & 0 & -1.1 & 0 & 0 \end{pmatrix},$$

$$d_{\mathrm{Left-DW}}^{\mathrm{interface}} = \begin{pmatrix} 1 & 1.1 & 1.1 & 0 & 0 & 1 \\ 1 & -1 & 0 & 0 & 0 & 1.1 \\ 0 & 0 & 0 & 0 & 1.1 & 0 \end{pmatrix},$$

$$d_{\mathrm{Right-DW}}^{\mathrm{interface}} = \begin{pmatrix} -1 & -1.1 & -1.1 & 0 & 0 & 1 \\ 1 & -1 & 0 & 0 & 0 & -1.1 \\ 0 & 0 & 0 & 0 & -1.1 & 0 \end{pmatrix},$$

$$d_{P_{\mathrm{up}}}^{\mathrm{interface}} = \begin{pmatrix} 0 & 0 & 0 & 0 & 1.1 & 1 \\ 1 & -1 & 0 & 1.1 & 0 & 0 \\ 1.1 & 1.1 & 1 & 0 & 0 & 0 \end{pmatrix},$$

$$d_{P_{\mathrm{down}}}^{\mathrm{interface}} = \begin{pmatrix} 0 & 0 & 0 & 0 & -1.1 & 1 \\ 1 & -1 & 0 & -1.1 & 0 & 0 \\ -1.1 & -1.1 & -1 & 0 & 0 & 0 \end{pmatrix}. \quad \text{(S3)}$$

The second harmonic polarization at 1L MoS_2/PZT interface, $\boldsymbol{P}_{\mathrm{interface}}^{2\omega}$, is expressed as:

$$\begin{pmatrix} P_x^{2\omega}(\varphi) \\ P_y^{2\omega}(\varphi) \\ P_z^{2\omega}(\varphi) \end{pmatrix} = \varepsilon_0 \left(d_{\mathrm{DW}}^{\mathrm{interface}} \right) \begin{pmatrix} E_x^2(\omega) \\ E_y^2(\omega) \\ E_z^2(\omega) \\ 2E_y(\omega)E_z(\omega) \\ 2E_x(\omega)E_z(\omega) \\ 2E_x(\omega)E_y(\omega) \end{pmatrix} \propto d_{\mathrm{DW}}^{\mathrm{interface}} \begin{pmatrix} cos^2\varphi \\ sin^2\varphi \\ 0 \\ 0 \\ 0 \\ -sin2\varphi \end{pmatrix}, \quad \text{(S4)}$$

where φ is the polarization angle of the incident light relative to *x*-axis, ε_0 is the vacuum permittivity, $E_x = E_0 \cos\varphi$, $E_y = E_0 \sin\varphi$, and $E_z = 0$ are the *x*-, *y*-, and *z*-component of the electric field of the fundamental wave. In our experiments, $\varphi = 0°$.

To model the SHG mapping results in Fig. 3e, we derived the explicit expressions of $d_{ij}^{\mathrm{interface}}$ at the four DWs as a function of the stacking angle (θ):

$$d_{\text{Top-DW}}^{\text{interface}}(\theta) =$$

$$\begin{pmatrix} -\sin^3(\theta)d_{33} - \sin(2\theta)\cos(\theta)\,d_{15} & -\cos^2(\theta)\sin(\theta)d_{33} + \cos(2\theta)\sin(\theta)d_{15} & -\sin(\theta)d_{15} & 0 & 0 & 2\sin^2(\theta)\cos(\theta)d_{33} + \cos(3\theta)d_{15} + d_{\text{MoS}_2} \\ \cos(\theta)\sin^2(\theta)d_{33} + [\cos^3(\theta) - \sin(\theta)\cos^2(\theta)]d_{15} + d_{\text{MoS}_2} & \cos^3(\theta)d_{33} + [\cos(\theta)\sin^2(\theta) - sin(\theta)\cos^2(\theta)]d_{15} - d_{\text{MoS}_2} & \cos(\theta)d_{15} & 0 & 0 & -2\cos^2(\theta)sin(\theta)d_{33} + \sin(3\theta)d_{15} \\ 0 & 0 & 0 & \cos(\theta)d_{15} & -\sin(\theta)d_{15} & 0 \end{pmatrix},$$

$$d_{\text{Bottom-DW}}^{\text{interface}}(\theta) =$$

$$\begin{pmatrix} -\sin^3(\theta)d_{33} + \sin(2\theta)\cos(\theta)\,d_{15} & \cos^2(\theta)\sin(\theta)d_{33} - \cos(2\theta)\sin(\theta)d_{15} & \sin(\theta)d_{15} & 0 & 0 & -\sin(2\theta)\sin(\theta)d_{33} - \cos(3\theta)d_{15} + d_{\text{MoS}_2} \\ -\cos(\theta)\sin^2(\theta)d_{33} - \cos(2\theta)\cos(\theta)d_{15} + d_{\text{MoS}_2} & -\cos^3(\theta)d_{33} - \sin(2\theta)\sin(\theta)d_{15} - d_{\text{MoS}_2} & -\cos(\theta)d_{15} & 0 & 0 & \sin(2\theta)\,cos(\theta)d_{33} - \sin(3\theta)d_{15} \\ 0 & 0 & 0 & -\cos(\theta)d_{15} & \sin(\theta)d_{15} & 0 \end{pmatrix},$$

$$d_{\text{Left-DW}}^{\text{interface}}(\theta) =$$

$$\begin{pmatrix} \cos^3(\theta)d_{33} + \sin(2\theta)\sin(\theta)d_{15} & \cos(\theta)\sin^2(\theta)d_{33} + \cos(2\theta)\cos(\theta)d_{15} & \cos(\theta)d_{15} & 0 & 0 & 2\cos^2(\theta)\sin(\theta)\,d_{33} - \sin(3\theta)d_{15} + d_{\text{MoS}_2} \\ \cos^2(\theta)\sin(\theta)d_{33} + [\sin^3(\theta) + cos(\theta)sin^2(\theta)]d_{15} + d_{\text{MoS}_2} & \sin^3(\theta)d_{33} + [\sin(\theta)\cos^2(\theta) + \cos(\theta)\sin^2(\theta)]\,d_{15} - d_{\text{MoS}_2} & \sin(\theta)d_{15} & 0 & 0 & 2\sin^2(\theta)cos(\theta)\,d_{33} + \cos(3\theta)d_{15} \\ 0 & 0 & 0 & \sin(\theta)d_{15} & \cos(\theta)d_{15} & 0 \end{pmatrix},$$

$$d_{\text{Right-DW}}^{\text{interface}}(\theta) =$$

$$\begin{pmatrix} -\cos^3(\theta)d_{33} - \sin(2\theta)\sin(\theta)d_{15} & -\cos(\theta)\sin^2(\theta)d_{33} - \cos(2\theta)\cos(\theta)d_{15} & -\cos(\theta)d_{15} & 0 & 0 & -2\cos^2(\theta)\sin(\theta)\,d_{33} + \sin(3\theta)d_{15} + d_{\text{MoS}_2} \\ -\cos^2(\theta)\sin(\theta)d_{33} - [\sin^3(\theta) + \cos(\theta)\sin^2(\theta)]d_{15} + d_{\text{MoS}_2} & -\sin^3(\theta)d_{33} - [\sin(\theta)\cos^2(\theta) + \cos(\theta)\sin^2(\theta)]d_{15} - d_{\text{MoS}_2} & -\sin(\theta)d_{15} & 0 & 0 & 2\sin^2(\theta)cos(\theta)\,d_{33} - \cos(3\theta)d_{15} \\ 0 & 0 & 0 & -\sin(\theta)d_{15} & -\cos(\theta)d_{15} & 0 \end{pmatrix}.$$

(S5)

Supplementary Table 2 lists the calculated interfacial SHG intensity at the DWs with different stacking angles θ between MoS_2 and the PZT domain structure, and the normalized experimental data extracted from Fig. 3e. The uncertainty of θ was estimated based on that of the MoS_2 transfer (1°) and domain writing (up to 5°), with the latter extracted from the PFM images in Fig. 3c. As shown in Fig. 3f and Supplementary Table 2, the simulated intensity distribution captures well the main features of the SHG tailoring pattern in the experimental results (Fig. 3e). Large deviation from the simulated values only occurs when the DW is in the vicinity of a cracked area in MoS_2, which compromises the net SHG signal.

Top-DW			Bottom-DW			Left-DW			Right-DW		
θ	Theory	Exp.	θ	Theory	Exp.	θ	Theory	Exp.	θ	Theory	Exp.
0°±2°	4.41±0.00	4.1±0.3	0°±2°	0.02±0.01	0.0±0.1	0°±2°	2.00±0.01	2.1±0.2	0°±2°	2.00±0.01	1.9±0.1
15°±2°	4.24±0.05	3.6±0.2	15°±2°	0.30±0.08	0.2±0.1	15°±6°	2.1±0.1	2.6±0.2	15°±5°	2.10±0.04	1.4±0.1
30°±2°	3.77±0.08	3.5±0.1	30°±2°	1.0±0.1	0.6±0.1	30°±4°	2.5±0.2	3.1±0.2	30°±5°	2.08±0.08	0.8±0.1
45°±2°	3.11±0.09	3.0±0.1	45°±3°	1.7±0.1	0.7±0.1	45°±6°	3.1±0.3	3.3±0.2	45°±6°	1.7±0.2	0.6±0.2

Supplementary Table 2 | Theory-experiment comparison for reflected SHG amplitude at 1L MoS_2/PZT DW. The experimental values are normalized to the average intensity difference between the Bottom-DW (set as 0) and vertical DWs (set as 2) at stacking angle $\theta = 0°$.

Supplementary References

1 Kumar, N. *et al.* Second harmonic microscopy of monolayer MoS_2. *Physical Review B* **87**, 161403 (2013).

2 Li, Y. L. *et al.* Probing Symmetry Properties of Few-Layer MoS_2 and h-BN by Optical Second-Harmonic Generation. *Nano Letters* **13**, 3329-3333 (2013).

3 Castellanos-Gomez, A. *et al.* Deterministic transfer of two-dimensional materials by all-dry viscoelastic stamping. *2D Materials* **1**, 011002 (2014).

4 Li, C. H., McCreary, K. M. & Jonker, B. T. Spatial Control of Photoluminescence at Room Temperature by Ferroelectric Domains in Monolayer WS_2/PZT Hybrid Structures. *ACS Omega* **1**, 1075-1080 (2016).

5 Wen, B. *et al.* Ferroelectric-Driven Exciton and Trion Modulation in Monolayer Molybdenum and Tungsten Diselenides. *ACS Nano* **13**, 5335-5343 (2019).

6 Kalinin, S. V. & Bonnell, D. A. Local potential and polarization screening on ferroelectric surfaces. *Physical Review B* **63**, 125411 (2001).

7 Song, J. F. *et al.* Enhanced Piezoelectric Response in Hybrid Lead Halide Perovskite Thin Films via Interfacing with Ferroelectric $PbZr_{0.2}Ti_{0.8}O_3$. *ACS Applied Materials & Interfaces* **10**, 19218-19225 (2018).

8 Kalinin, S. V., Johnson, C. Y. & Bonnell, D. A. Domain polarity and temperature induced potential inversion on the $BaTiO_3$ (100) surface. *Journal of Applied Physics* **91**, 3816-3823 (2002).

9 Hong, X. *et al.* Unusual resistance hysteresis in n-layer graphene field effect transistors fabricated on ferroelectric $Pb(Zr_{0.2}Ti_{0.8})O_3$. *Applied Physics Letters* **97**, 033114 (2010).

10 Giannozzi, P. *et al.* QUANTUM ESPRESSO: a modular and open-source software project for quantum simulations of materials. *Journal of physics: Condensed matter* **21**, 395502 (2009).

11 Vanderbilt, D. Soft self-consistent pseudopotentials in a generalized eigenvalue formalism. *Physical Review B* **41**, 7892 (1990).

12 Perdew, J. P. & Zunger, A. Self-interaction correction to density-functional approximations for many-electron systems. *Physical Review B* **23**, 5048 (1981).

13 Vaz, C. A. F. *et al.* Origin of the Magnetoelectric Coupling Effect in $Pb(Zr_{0.2}Ti_{0.8})O_3/La_{0.8}Sr_{0.2}MnO_3$ Multiferroic Heterostructures. *Physical Review Letters* **104**, 127202 (2010).

14 Zhang, L. *et al.* Effect of strain on ferroelectric field effect in strongly correlated oxide $Sm_{0.5}Nd_{0.5}NiO_3$. *Applied Physics Letters* **107**, 152906 (2015).

15 Jia, C.-L., Urban, K. W., Alexe, M., Hesse, D. & Vrejoiu, I. Direct Observation of Continuous Electric Dipole Rotation in Flux-Closure Domains in Ferroelectric $Pb(Zr,Ti)O_3$. *Science* **331**, 1420-1423 (2011).

16 King-Smith, R. & Vanderbilt, D. Theory of polarization of crystalline solids. *Physical Review B* **47**, 1651 (1993).

17 Hlinka, J. & Marton, P. Phenomenological model of a 90 degrees domain wall in $BaTiO_3$-type ferroelectrics. *Physical Review B* **74**, 104104 (2006).

18 Cherifi-Hertel, S. *et al.* Non-Ising and chiral ferroelectric domain walls revealed by nonlinear optical microscopy. *Nature Communications* **8**, 15768 (2017).

19 Hermet, P., Veithen, M. & Ghosez, P. Raman scattering intensities in $BaTiO_3$ and $PbTiO_3$ prototypical ferroelectrics from density functional theory. *Journal of Physics-Condensed Matter* **21**, 215901 (2009).
